# Introduction to *Toxins* Special Issue on Botulinum Toxins: New Uses in the Treatment of Diseases (2nd Edition)

**DOI:** 10.3390/toxins17100485

**Published:** 2025-09-28

**Authors:** Siro Luvisetto

**Affiliations:** National Research Council of Italy—CNR, Institute of Biochemistry and Cell Biology (IBBC), Via Ercole Ramarini 32, Monterotondo Scalo, 00015 Roma, Italy; siro.luvisetto@cnr.it

*Clostridium botulinum* strains produce seven antigenically distinct botulinum neurotoxins (BoNTs), referred to as serotypes A–G, and a hybrid toxin called F/A. All of these serotypes inhibit neurotransmission by blocking the release of synaptic neurotransmitters through the cleavage of specific sites on SNARE proteins. Serotypes A (BoNT/A) and B (BoNT/B) are widely used in the treatment of various movement and autonomic disorders in humans, with new indications continuing to increase. This Special Issue (SI), titled “Botulinum Neurotoxins: New Uses in the Treatment of Diseases (2nd Edition)”, is the second edition of a previous SI published in 2023 [1]. This SI is a collection of 13 research and review articles, 3 focusing on animals and 10 on humans, dedicated to novel applications of BoNTs in conditions where they have shown beneficial effects and potential as therapeutics for approval in the future.

The first article, by Jung et al. [2], reports a study with great novelty on the effects of BoNT serotype E (BoNT/E) in three pain models in rats: orofacial formalin-induced pain, complete Freund’s adjuvant (CFA)-induced thermal hyperalgesia, and neuropathic pain caused by inferior alveolar nerve injury in rats. The authors prove that subcutaneous BoNT/E injection significantly reduces nocifensive behaviour in response to formalin pain, alleviates thermal hypersensitivity in the CFA test, and attenuates mechanical allodynia after nerve injury. Furthermore, in parallel with the antiallodynic effects, the authors evidenced a reduced number of neurons expressing c-fos in the caudal trigeminal subnucleus. Overall, these results suggest that BoNT/E, like BoNT/A, should be considered a potential therapy for chronic pain, thus stimulating further studies for its potential future application in humans.

In the next article, Marinelli et al. [3] analysed the interaction of BoNT/A and BoNT/B with the neurotransmitter systems involved in formalin-induced inflammatory pain in mice. Unlike in Jung et al. [2], in which orofacial pain was induced by formalin, the authors of this study injected formalin into the hind paw of the mouse. It should be noted here that formalin injection into the hind paw induces a biphasic behavioural response characterised by a short temporal phase of intense licking of the injected paw, followed by an interphase of relative quiescence, and finally by a second prolonged phase of intense licking activity. It has previously been shown that BoNT/A reduces the second phase while BoNT/B abolishes the interphase. Thanks to the pharmacological interaction with glutamatergic antagonists and GABAergic agonists, the authors proved that BoNT/A acts mainly on excitatory synapses, while BoNT/B acts mainly on inhibitory synapses, thus contributing to improved understanding of the mechanisms of BoNTs’ interaction with pain pathways.

In another study, Seo et al. [4] analysed the effect of BoNT/A administration on nerve regeneration processes after sciatic nerve injury in rats. BoNT/A was injected directly into the site of nerve injury at two different time points: immediately or one week after the injury. Nerve regeneration was assessed several weeks after injury by measuring both the levels of various proteins involved in regeneration processes, the muscle action potential, and the sciatic function index. After BoNT/A injection, both immediately and one week after injury, increased expression of glial fibrillary acidic protein, astroglia calcium-binding protein, growth-associated protein 43, neurofilament 200, and brain-derived neurotrophic factor was seen. Electrophysiological and functional analyses showed that BoNT/A administration immediately after injury is most effective for neural recovery, although significant recovery was also seen during the non-acute phase following nerve damage.

Moving on to human studies, but remaining in the field of pain, Tamayo et al. [5] performed a meta-analysis on the effects of BoNT/A in pain-related post-stroke spasticity (pPSS). Specifically, the authors analysed the effects of four different commercial toxins (*AbobotulinumtoxinA, IncobotulinumtoxinA, Neu-BoNT-A*, and *OnabotulinumtoxinA*) used both as an early treatment within 12 weeks of stroke and as a late treatment more than 12 weeks after stroke. Their data analysis suggested that as an early treatment, BoNT-A is no more effective in reducing pPSS than a placebo. Conversely, BoNT-A proved to be preferable to a placebo as a late treatment for pPSS management. On the same topic, Bianchi et al. [6] performed a systematic review on the clinical effectiveness of *onabotulinumtoxinA* in pain control in adult patients with spasticity. Although the heterogeneity of the studies analysed did not allow for statistical analysis, the published evidence is consistent with an overall positive trend for the use of *onabotulinumtoxinA* in reducing spasticity-related pain in adults. The article by Deniz et al. [7] addresses another aspect of spasticity, presenting a retrospective study on the efficacy of BoNT/A associated with shock wave therapy on spasticity in patients with brain lesions. Specifically, considering that extracorporeal shock wave therapy (ESWT) is a safe, effective, and non-invasive treatment for spasticity, the authors compared the effect of unfocused ESWT (uESWT) with the more widely used focal ESWT (fESWT). The treatment of patients was performed using *IncobotulinumtoxinA* in the upper limb or *abobotulinumtoxinA* in the lower limb. The authors show that uESWT combined with BoNT-A has a similar effect in patients with stroke and multiple sclerosis to that achieved with fESWT and BoNT-A, but with higher patient satisfaction reported for uESWT.

Next, a series of review articles on the effect of BoNTs on movement disorders, such as Parkinson’s disease and cervical dystonia, are presented. De Souza et al. [8] present a retrospective analysis on the association between BoNTs and deep brain stimulation (DBS) in patients with generalised dystonia. This study shows that, compared to increasing the BoNT dosage over time in dystonia patients not undergoing DBS, the combination of BoNTs with DBS results in a reduction in subsequent BoNT dosages. In another review, Das and Jog [9] analysed the effects of peripheral BoNT/A injection on sensorimotor integration (SMI), with particular focus on the pathways through which it may act, in conditions such as Parkinson’s disease (PD), cervical dystonia (CD), and writer’s cramp (WC), in which SMI is impaired. The authors concluded that BoNT/A alters the afferent input from muscle spindles and sensory receptors, thereby influencing spinal and cortical circuits and causing changes in SMI and brain plasticity. In addition to its peripheral effect, this mechanism may contribute to the beneficial effect of BoNT/A in the treatment of PD, CD and WC. Finally, a review from Popescu et al. [10] focuses on BoNT/A injection into the masseter muscle as a treatment for a range of medical conditions, including hypertrophy, bruxism, temporomandibular and oromandibular disorders, myofascial pain syndrome, and also for aesthetic purposes to correct facial disharmony. Despite the well-known beneficial effects of BoNT/A in a number of human conditions, the authors highlight the potential risks associated with injection into the masseter muscle, which, due to the possible diffusion of the toxin, may cause relaxation in the muscles adjacent to the injection site. Furthermore, because toxin placement based exclusively on anatomical references is highly imprecise and can lead to unwanted side effects, the authors recommend the use of ultrasound guidance to improve the injection’s accuracy and safety.

An interesting new indication for therapeutic use of BoNT/A is proposed by Cosentino et al. [11]. The authors present an open-label prospective study in which an electromyography-guided unilateral injection of *onabotulinumtoxinA* is suggested as a safe treatment for dysfunction of the retrograde cricopharyngeal muscle, a condition characterised by the inability to burp, typically accompanied by gurgling, bloating, and flatulence. The results reported in this study prove a sustained reduction in the severity of symptoms, with significant improvements already evident after 1 month and maintained for at least 4 months. Furthermore, the authors present a correlation between the electromyographic parameters of the cricopharyngeal muscle with symptomatic burden and response to treatment.

In a narrative review, O’Donhoe et al. [12] explore the minimum effective dosages, optimal administration techniques, and pharmacological safety profile of BoNT/A in the treatment of Raynaud’s syndrome of the hand, a generalised condition characterised by recurrent episodic vasospasm of the fingers, accompanied by pallor, cyanosis, and pain. The authors analyse several studies investigating the clinical efficacy of subcutaneous BoNT/A administration, highlighting its clinical efficacy using both patient-reported outcome measures and objective outcome measures from perfusion, thermography, and pulsed pressure studies.

Another review is presented by Gaseminejad-Bandpey et al. [13], who analyse data on BoNT therapy in psoriasis and explore its potential role of a treatment for this medical disorder. Fifteen relevant articles, eleven on human and four on animal studies, were analysed. Specifically, eight of the human studies were open-label clinical trials and three were single case reports. A general improvement in psoriasis was observed after intradermal or subcutaneous BoNT/A or BoNT/B injections. On the other hand, animal studies in psoriatic mouse models have shown that BoNT injection into the skin heals psoriatic skin lesions and reduces the level of interleukins and cytokines as well as inflammatory cells in psoriatic plaques. Given the limitations of open-label studies and case reports, the authors conclude by calling for controlled, blinded studies on a larger number of patients, using doses that have shown promise in open-label studies.

Finally, Perez et al. [14] review all recent studies on BoNT treatments for hair and scalp disorders. They evaluate the clinical efficacy of using BoNT/A or BoNT/B in the treatment of hair and scalp disorders, such as hair loss, scalp seborrheic dermatitis/hyperseborrhea, craniofacial hyperhidrosis, folliculitis decalvans/folliculitis dissecans, scalp pain, and linear scleroderma. The authors conclude that most studies on BoNT therapy for androgenetic alopecia report mild or insignificant hair growth, while they demonstrate some efficacy in the treatment of craniofacial hyperhidrosis with minimal side effects. Currently, it is difficult to draw definitive and clear conclusions on the efficacy of BoNTs in the treatment of hair and scalp disorders, given the wide variability in outcome measures; thus, larger, more controlled studies are required.

In conclusion, the contributions to this *Toxins* SI contribute to the advancement of knowledge on novel therapeutic uses of BoNTs. As many of the published studies are dedicated to emerging applications of BoNTs in uncommon diseases, this SI provides new data to support a better understanding of the use of BoNTs for improving human health.
